# 4,5,6,7-Tetra­chloro-2-(2,2,2-trifluoro­eth­yl)isoindoline-1,3-dione

**DOI:** 10.1107/S1600536810023019

**Published:** 2010-06-23

**Authors:** Xian-Shu Fu, Xiao-Ping Yu, Wei-Min Wang, Fang Lin

**Affiliations:** a College of Life Sciences, China Jiliang University, Hangzhou 310018, People’s Republic of China

## Abstract

In the title compound, C_10_H_2_Cl_4_F_3_NO_2_, the isoindoline ring system is almostplanar, the maximum atomic deviation being 0.064 (2) Å. The C—C bond of the ethyl­ene group is twisted with respect to the isoindoline plane by a dihedral angle of 59.58 (12)°. In the crystal, weak inter­molecular C—H⋯F hydrogen bonding links the mol­ecules into supra­molecular chains running along the *a* axis. A short inter­molecular Cl⋯O contact of 2.950 (3) Å is also observed.

## Related literature

The title compound is an inter­mediate in the synthesis of organic electro-luminescent materials, see: Han & Kay (2005 ▶). For a related structure, see: Valkonen *et al.* (2007 ▶).
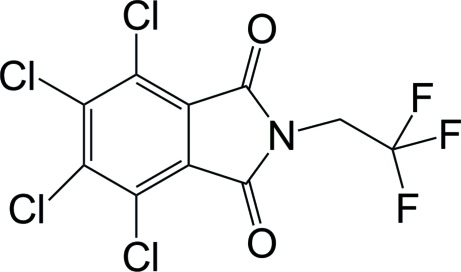


## Experimental

### 

#### Crystal data

C_10_H_2_Cl_4_F_3_NO_2_
*M* *_r_* = 366.93
Triclinic, 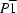

*a* = 4.943 (4) Å
*b* = 10.759 (9) Å
*c* = 12.130 (11) Å
α = 101.373 (19)°
β = 101.18 (2)°
γ = 92.704 (3)°
*V* = 617.9 (9) Å^3^
*Z* = 2
Mo *K*α radiation
μ = 0.99 mm^−1^
*T* = 113 K
0.20 × 0.08 × 0.06 mm

#### Data collection

Rigaku Saturn CCD area-detector diffractometer
Absorption correction: multi-scan (*CrystalClear*; Rigaku/MSC, 2001 ▶) *T*_min_ = 0.826, *T*_max_ = 0.943
5139 measured reflections
2126 independent reflections
2033 reflections with *I* > 2σ(*I*)
*R*_int_ = 0.027

#### Refinement

*R*[*F* ^2^ > 2σ(*F* ^2^)] = 0.027
*wR*(*F* ^2^) = 0.078
*S* = 1.07
2126 reflections
181 parameters
H-atom parameters constrained
Δρ_max_ = 0.33 e Å^−3^
Δρ_min_ = −0.34 e Å^−3^

### 

Data collection: *CrystalClear* (Rigaku/MSC, 2001 ▶); cell refinement: *CrystalClear*; data reduction: *CrystalStructure* (Rigaku/MSC, 2004 ▶); program(s) used to solve structure: *SHELXS97* (Sheldrick, 2008 ▶); program(s) used to refine structure: *SHELXL97* (Sheldrick, 2008 ▶); molecular graphics: *SHELXTL* (Sheldrick, 2008 ▶); software used to prepare material for publication: *SHELXL97*.

## Supplementary Material

Crystal structure: contains datablocks I, global. DOI: 10.1107/S1600536810023019/xu2778sup1.cif

Structure factors: contains datablocks I. DOI: 10.1107/S1600536810023019/xu2778Isup2.hkl

Additional supplementary materials:  crystallographic information; 3D view; checkCIF report

## Figures and Tables

**Table 1:** Hydrogen-bond geometry (Å, °)

*D*—H⋯*A*	*D*—H	H⋯*A*	*D*⋯*A*	*D*—H⋯*A*
C9—H9*B*⋯F1^i^	0.99	2.38	3.289 (4)	152

Symmetry code: (i) 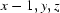.

**Fig. 1.**
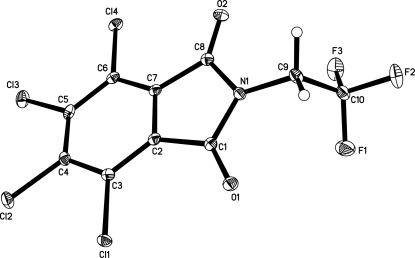
View of the molecule of showing the atom-labelling scheme. Displacement ellipsoids are drawn at the 30% probability level.

## supplementary crystallographic information

### Comment

The title compound is a key intermediate in the synthesis of organic electro-luminescent materials. The emission of light by organic molecules exposed to an electric field has been wide investigated in both an academic and industrial context (Han & Kay, 2005).

The molecular structure of the title compound is illustrated in Fig. 1. The isoindole ring system is planar, the maximum atomic deviation being 0.064 (2)Å (for C8 atom). The C9—C10 bond of the ethylene group is twisted with respect to the isoindole ring by a dihedral angle of 59.58 (12)°, which is similar to 60.3 (5)° found in a related compound 2-(2-iodoethyl)isoindole-1,3-dione (Valkonen *et al.* 2007). Weak intermolecular C—H···F hydrogen bonding is present in the crystal structure (Table 1).

### Experimental

An acetic acid solution of tetrachlorophthalic anhydride (28.6 g, 100 mmol) and 2,2,2-trifluoroethylamine (7.99 ml, 100 mmol) was refluxed overnight, and then filtered. The crude produce was recrystallized from ethyl acetate.

### Refinement

H atoms were positioned geometrically and refined as riding with C—H = 0.99 Å, and *U*_iso_(H) = 1.2*U*_eq_(C).

### Crystal data

**Table:**

C_10_H_2_Cl_4_F_3_NO_2_	*Z* = 2
*M**_r_* = 366.93	*F*(000) = 360
Triclinic, *P*1	*D*_x_ = 1.972 Mg m^−^^3^
Hall symbol: -P 1	Melting point: 477 K
*a* = 4.943 (4) Å	Mo *K*α radiation, λ = 0.71073 Å
*b* = 10.759 (9) Å	Cell parameters from 2510 reflections
*c* = 12.130 (11) Å	θ = 1.8–27.9°
α = 101.373 (19)°	µ = 0.99 mm^−^^1^
β = 101.18 (2)°	*T* = 113 K
γ = 92.704 (3)°	Prism, colorless
*V* = 617.9 (9) Å^3^	0.20 × 0.08 × 0.06 mm

### Data collection

**Table:**

Rigaku Saturn CCD area-detector diffractometer	2126 independent reflections
Radiation source: fine-focus sealed tube	2033 reflections with *I* > 2σ(*I*)
graphite	*R*_int_ = 0.027
Detector resolution: 14.63 pixels mm^-1^	θ_max_ = 25.0°, θ_min_ = 1.8°
ω and φ scans	*h* = −5→5
Absorption correction: multi-scan (*CrystalClear*; Rigaku/MSC, 2001)	*k* = −12→12
*T*_min_ = 0.826, *T*_max_ = 0.943	*l* = −14→11
5139 measured reflections	

### Refinement

**Table:**

Refinement on *F*^2^	Primary atom site location: structure-invariant direct methods
Least-squares matrix: full	Secondary atom site location: difference Fourier map
*R*[*F*^2^ > 2σ(*F*^2^)] = 0.027	Hydrogen site location: inferred from neighbouring sites
*wR*(*F*^2^) = 0.078	H-atom parameters constrained
*S* = 1.07	*w* = 1/[σ^2^(*F*_o_^2^) + (0.0516*P*)^2^ + 0.2268*P*] where *P* = (*F*_o_^2^ + 2*F*_c_^2^)/3
2126 reflections	(Δ/σ)_max_ = 0.003
181 parameters	Δρ_max_ = 0.33 e Å^−^^3^
0 restraints	Δρ_min_ = −0.34 e Å^−^^3^

### Special details

**Table:**

Geometry. All e.s.d.'s (except the e.s.d. in the dihedral angle between two l.s. planes) are estimated using the full covariance matrix. The cell e.s.d.'s are taken into account individually in the estimation of e.s.d.'s in distances, angles and torsion angles; correlations between e.s.d.'s in cell parameters are only used when they are defined by crystal symmetry. An approximate (isotropic) treatment of cell e.s.d.'s is used for estimating e.s.d.'s involving l.s. planes.
Refinement. Refinement of *F*^2^ against ALL reflections. The weighted *R*-factor *wR* and goodness of fit *S* are based on *F*^2^, conventional *R*-factors *R* are based on *F*, with *F* set to zero for negative *F*^2^. The threshold expression of *F*^2^ > σ(*F*^2^) is used only for calculating *R*-factors(gt) *etc*. and is not relevant to the choice of reflections for refinement. *R*-factors based on *F*^2^ are statistically about twice as large as those based on *F*, and *R*- factors based on ALL data will be even larger.

### Fractional atomic coordinates and isotropic or equivalent isotropic displacement parameters (Å^2^)

**Table:**

	*x*	*y*	*z*	*U*_iso_*/*U*_eq_	
Cl1	0.45684 (8)	0.62343 (4)	0.10599 (3)	0.01714 (14)	
Cl2	0.43932 (10)	0.86631 (4)	0.29132 (4)	0.02492 (14)	
Cl3	0.10966 (10)	0.85656 (4)	0.48243 (4)	0.02680 (15)	
Cl4	−0.26190 (9)	0.61244 (4)	0.47757 (4)	0.01965 (14)	
F1	0.1532 (2)	0.08394 (12)	0.15299 (12)	0.0368 (3)	
F2	−0.2343 (3)	−0.03074 (10)	0.09326 (10)	0.0347 (3)	
F3	−0.1393 (3)	0.08654 (11)	0.26249 (10)	0.0356 (3)	
O1	0.1734 (2)	0.34347 (11)	0.05198 (10)	0.0189 (3)	
O2	−0.4074 (2)	0.34517 (12)	0.30361 (11)	0.0209 (3)	
N1	−0.1296 (3)	0.31251 (13)	0.16870 (12)	0.0157 (3)	
C1	0.0657 (3)	0.38289 (16)	0.13115 (15)	0.0145 (3)	
C2	0.1009 (3)	0.51129 (16)	0.20884 (14)	0.0140 (3)	
C3	0.2573 (3)	0.61999 (16)	0.20684 (14)	0.0148 (3)	
C4	0.2506 (3)	0.72884 (15)	0.29118 (15)	0.0165 (4)	
C5	0.0966 (4)	0.72557 (16)	0.37583 (14)	0.0177 (4)	
C6	−0.0662 (3)	0.61525 (16)	0.37516 (15)	0.0155 (3)	
C7	−0.0635 (3)	0.50918 (16)	0.28992 (14)	0.0143 (3)	
C8	−0.2256 (3)	0.38237 (16)	0.26155 (14)	0.0155 (4)	
C9	−0.2519 (4)	0.18735 (16)	0.10544 (15)	0.0190 (4)	
H9A	−0.2347	0.1774	0.0243	0.023*	
H9B	−0.4515	0.1801	0.1069	0.023*	
C10	−0.1160 (4)	0.08224 (17)	0.15460 (15)	0.0196 (4)	

### Atomic displacement parameters (Å^2^)

**Table:**

	*U*^11^	*U*^22^	*U*^33^	*U*^12^	*U*^13^	*U*^23^
Cl1	0.0164 (2)	0.0208 (2)	0.0167 (2)	0.00139 (17)	0.00554 (17)	0.00765 (17)
Cl2	0.0304 (3)	0.0152 (2)	0.0292 (3)	−0.00270 (18)	0.0068 (2)	0.00546 (18)
Cl3	0.0384 (3)	0.0184 (2)	0.0217 (3)	0.00406 (19)	0.0080 (2)	−0.00242 (18)
Cl4	0.0217 (2)	0.0254 (2)	0.0152 (2)	0.00822 (18)	0.00803 (17)	0.00679 (17)
F1	0.0242 (6)	0.0350 (7)	0.0603 (9)	0.0079 (5)	0.0129 (6)	0.0260 (6)
F2	0.0502 (7)	0.0160 (5)	0.0319 (7)	−0.0055 (5)	0.0022 (5)	−0.0006 (5)
F3	0.0617 (8)	0.0289 (6)	0.0198 (6)	0.0048 (6)	0.0135 (5)	0.0085 (5)
O1	0.0202 (6)	0.0203 (6)	0.0168 (6)	0.0020 (5)	0.0072 (5)	0.0022 (5)
O2	0.0175 (6)	0.0244 (7)	0.0233 (7)	−0.0005 (5)	0.0089 (5)	0.0066 (5)
N1	0.0158 (7)	0.0148 (7)	0.0163 (7)	−0.0005 (6)	0.0040 (6)	0.0024 (6)
C1	0.0138 (8)	0.0155 (8)	0.0144 (8)	0.0028 (6)	0.0005 (6)	0.0056 (6)
C2	0.0129 (8)	0.0165 (8)	0.0131 (8)	0.0041 (6)	0.0011 (6)	0.0054 (6)
C3	0.0139 (8)	0.0187 (8)	0.0131 (8)	0.0031 (6)	0.0024 (6)	0.0066 (6)
C4	0.0177 (8)	0.0144 (8)	0.0176 (9)	0.0017 (7)	0.0006 (7)	0.0065 (7)
C5	0.0218 (9)	0.0157 (8)	0.0147 (9)	0.0070 (7)	0.0007 (7)	0.0026 (7)
C6	0.0154 (8)	0.0202 (8)	0.0127 (8)	0.0063 (7)	0.0033 (6)	0.0062 (7)
C7	0.0129 (8)	0.0174 (8)	0.0138 (8)	0.0049 (6)	0.0020 (6)	0.0065 (6)
C8	0.0148 (8)	0.0176 (8)	0.0146 (8)	0.0030 (6)	0.0018 (7)	0.0058 (7)
C9	0.0189 (9)	0.0174 (8)	0.0183 (9)	−0.0035 (7)	0.0018 (7)	0.0014 (7)
C10	0.0230 (9)	0.0179 (8)	0.0178 (9)	−0.0037 (7)	0.0065 (7)	0.0024 (7)

### Geometric parameters (Å, °)

**Table:**

Cl1—C3	1.719 (2)	C1—C2	1.492 (3)
Cl2—C4	1.711 (2)	C2—C3	1.378 (3)
Cl3—C5	1.704 (2)	C2—C7	1.395 (3)
Cl4—C6	1.720 (2)	C3—C4	1.401 (3)
F1—C10	1.334 (2)	C4—C5	1.396 (3)
F2—C10	1.334 (2)	C5—C6	1.400 (3)
F3—C10	1.328 (2)	C6—C7	1.383 (3)
O1—C1	1.202 (2)	C7—C8	1.492 (3)
O2—C8	1.205 (2)	C9—C10	1.506 (3)
N1—C1	1.397 (2)	C9—H9A	0.9900
N1—C8	1.402 (2)	C9—H9B	0.9900
N1—C9	1.450 (2)		
			
C1—N1—C8	113.24 (14)	C5—C6—Cl4	120.78 (14)
C1—N1—C9	122.43 (15)	C6—C7—C2	121.03 (16)
C8—N1—C9	123.58 (14)	C6—C7—C8	130.65 (16)
O1—C1—N1	125.00 (16)	C2—C7—C8	108.26 (15)
O1—C1—C2	130.05 (16)	O2—C8—N1	125.42 (16)
N1—C1—C2	104.95 (15)	O2—C8—C7	129.62 (16)
C3—C2—C7	121.73 (16)	N1—C8—C7	104.90 (14)
C3—C2—C1	129.80 (17)	N1—C9—C10	112.25 (15)
C7—C2—C1	108.46 (15)	N1—C9—H9A	109.2
C2—C3—C4	117.61 (17)	C10—C9—H9A	109.2
C2—C3—Cl1	121.85 (14)	N1—C9—H9B	109.2
C4—C3—Cl1	120.54 (14)	C10—C9—H9B	109.2
C5—C4—C3	120.88 (16)	H9A—C9—H9B	107.9
C5—C4—Cl2	119.90 (13)	F3—C10—F1	107.19 (15)
C3—C4—Cl2	119.21 (14)	F3—C10—F2	106.91 (15)
C4—C5—C6	120.82 (16)	F1—C10—F2	107.04 (15)
C4—C5—Cl3	119.78 (14)	F3—C10—C9	112.72 (16)
C6—C5—Cl3	119.40 (15)	F1—C10—C9	112.66 (15)
C7—C6—C5	117.84 (17)	F2—C10—C9	109.98 (16)
C7—C6—Cl4	121.38 (14)		
			
C8—N1—C1—O1	−178.89 (16)	Cl3—C5—C6—Cl4	2.3 (2)
C9—N1—C1—O1	−8.4 (3)	C5—C6—C7—C2	1.4 (2)
C8—N1—C1—C2	0.56 (18)	Cl4—C6—C7—C2	−178.43 (12)
C9—N1—C1—C2	171.02 (14)	C5—C6—C7—C8	−175.42 (16)
O1—C1—C2—C3	3.2 (3)	Cl4—C6—C7—C8	4.7 (3)
N1—C1—C2—C3	−176.17 (16)	C3—C2—C7—C6	−3.0 (2)
O1—C1—C2—C7	−178.26 (17)	C1—C2—C7—C6	178.38 (14)
N1—C1—C2—C7	2.33 (17)	C3—C2—C7—C8	174.49 (15)
C7—C2—C3—C4	1.3 (2)	C1—C2—C7—C8	−4.16 (18)
C1—C2—C3—C4	179.68 (15)	C1—N1—C8—O2	174.66 (16)
C7—C2—C3—Cl1	−179.20 (12)	C9—N1—C8—O2	4.3 (3)
C1—C2—C3—Cl1	−0.9 (3)	C1—N1—C8—C7	−3.00 (17)
C2—C3—C4—C5	1.7 (2)	C9—N1—C8—C7	−173.33 (14)
Cl1—C3—C4—C5	−177.73 (12)	C6—C7—C8—O2	4.0 (3)
C2—C3—C4—Cl2	−179.49 (12)	C2—C7—C8—O2	−173.14 (17)
Cl1—C3—C4—Cl2	1.0 (2)	C6—C7—C8—N1	−178.48 (17)
C3—C4—C5—C6	−3.3 (3)	C2—C7—C8—N1	4.39 (17)
Cl2—C4—C5—C6	177.95 (13)	C1—N1—C9—C10	99.37 (19)
C3—C4—C5—Cl3	175.92 (13)	C8—N1—C9—C10	−91.2 (2)
Cl2—C4—C5—Cl3	−2.8 (2)	N1—C9—C10—F3	61.1 (2)
C4—C5—C6—C7	1.7 (2)	N1—C9—C10—F1	−60.4 (2)
Cl3—C5—C6—C7	−177.55 (12)	N1—C9—C10—F2	−179.68 (14)
C4—C5—C6—Cl4	−178.51 (12)		

### Hydrogen-bond geometry (Å, °)

**Table:**

*D*—H···*A*	*D*—H	H···*A*	*D*···*A*	*D*—H···*A*
C9—H9B···F1^i^	0.99	2.38	3.289 (4)	152

Symmetry codes: (i) *x*−1, *y*, *z*.
